# An automated system for the objective evaluation of human gustatory sensitivity using tongue biopotential recordings

**DOI:** 10.1371/journal.pone.0177246

**Published:** 2017-08-02

**Authors:** Danilo Pani, Ilenia Usai, Piero Cosseddu, Melania Melis, Giorgia Sollai, Roberto Crnjar, Iole Tomassini Barbarossa, Luigi Raffo, Annalisa Bonfiglio

**Affiliations:** 1 Department of Electrical and Electronic Engineering, University of Cagliari, Cagliari, Italy; 2 Department of Biomedical Sciences, University of Cagliari, Monserrato, Italy; The University of Tokyo, JAPAN

## Abstract

The goal of this work is to develop an automatic system for the evaluation of the gustatory sensitivity of patients using an electrophysiological recording of the response of bud cells to taste stimuli. In particular, the study aims to evaluate the effectiveness and limitations of supervised classifiers in the discrimination between subjects belonging to the three 6-*n*-propylthiouracil (PROP) taster categories (supertasters, medium tasters, and non-tasters), exploiting features extracted from electrophysiological recordings of the tongue. Thirty-nine subjects (equally divided into the three PROP status classes by standard non-objective scaling methods) underwent a non-invasive, differential, biopotential recording of their tongues during stimulation with PROP by using a custom-made, flexible, silver electrode. Two different classifiers were trained to recognize up to seven different features extracted from the recorded depolarization signal. The classification results indicate that the identified set of features allows to distinguish between PROP tasters and non-tasters (average accuracy of 80% ± 18% and up to 94% ± 15% when only supertasters and non-tasters are considered), but medium tasters were difficult to identify. However, these apparent classification errors are related to uncertainty in the labeling procedures, which are based on non-objective tests, in which the subjects provided borderline evaluations. Thus, using the proposed method, it is possible, for the first time, to automatically achieve objective PROP taster status identification with high accuracy. The simplicity of the recording technique allows for easy reproduction of the experimental setting; thus the technique can be used in future studies to evaluate other gustatory stimuli. The proposed approach represents the first objective and automatic method to directly measure human gustatory responses and a milestone for physiological taste studies, with applications ranging from basic science to food tasting evaluations.

## Introduction

Taste perception varies from person to person, influencing food choices and habits [1]. Although there are five clearly recognizable basic tastes (sweet, sour, salt, bitter, and umami), several physiological studies have focused on the use of a bitter substance, 6-*n*-propylthiouracil (PROP), or its homologue phenylthiocarbamide (PTC), in order to evaluate the taste perception ability in humans [1,2]. This approach is based on data that indicate that PROP tasting is associated with variations in taste perception for various oral stimuli, including other bitter compounds [3], chemical irritants [4], and fats [5]. These studies have demonstrated how PROP taster status is related to ethnicity, sex, and age [1,6,7]. About 25%–30% of the Caucasian population does not perceive PROP taste (non-tasters), whereas the remaining does (tasters) [8]. Among the tasters, supertasters perceive PROP bitterness at levels far above the average (according to qualitative tests), whereas as their name suggests, medium tasters (MTs) occupy the middle ground between supertasters (STs) and non-tasters (NTs) [7,9].

A multiplicity of genetic and environmental factors have been shown to influence PROP perception [10,11]. Originally, it was thought that the *taste receptor 2 member 38* (*TAS2R38*, a bitter taste receptor) gene codes for the protein of the taste cell membrane that can bind to the N-C = S moiety of PROP, which is responsible for the bitter taste of PROP [8,12]. However, at present, we know that PROP tasting ability is related to two common different haplotypes of *TAS2R38*, the proline–alanine–valine (PAV) variant and the alanine–valine–isoleucine (AVI) variant, resulting from the single-nucleotide polymorphisms (SNPs): *rs713598*, *rs1726866*, and *rs10246939* [13,14]. The PAV variant is dominant and associated with the taster phenotype, whereas the AVI variant is recessive and associated with the NT phenotype. Tasters possess the PAV/PAV or PAV/AVI diplotype, whereas NTs are homozygous for the recessive haplotype (AVI/AVI); however, other uncommon haplotypes also exist (e.g., AAV, AAI, PVI, and PAI) [15]. The allelic diversity of the *TAS2R38* gene is mainly responsible for PROP taste variability, explaining 55%–80% of the PROP phenotype variance [13,14]. However, some studies have reported considerable genotypic overlap between the MT and ST groups [14,16].

STs have also been shown to possess a higher density of tongue papillae compared with NTs [7], so the PROP tasting ability also correlates with papilla density and morphology [17]. A factor that accounts for this difference is a polymorphism in the gene that codifies for a salivary protein secreted by parotid glands, gustin (carbonic anhydrase 6), which has been shown to affect PROP sensitivity by acting on cell growth and the maintenance of fungiform papillae, thereby providing an explanation as to why PROP STs are more responsive to a broad range of stimuli [17]. Moreover, oral sensitivity to PROP, or its homologue PTC, has been related to other modifying genes [18,19] and to the expression of specific salivary amino acids and/or proteins [20–22].

However, scientific literature in the field reports inconsistent data either on the role of some of these factors in PROP tasting [23,24] or on the role that this trait plays in oral taste perception and food preferences with corresponding health implications, which suggests the presence of confounding variables [25–27]. Thus, there is a great need in taste sensitivity studies for methods that allow an objective measure capable of effectively assessing the activation level of the gustatory system. In fact, past studies tend to rely on psychophysical approaches, which, despite their straightforward implementation, produce highly subjective evaluations and measurement errors that may account for up to 20% of the phenotypic variance [23]. In a previous work [28], a novel non-invasive technique for the direct measurement of the degree of activation of peripheral taste function in humans through electrophysiological recordings was presented. The efficacy of that technique was demonstrated by the relationship between amplitude and rate of the signal and subjects’ PROP genotype and phenotype.

Based on the evidence of such a seminal work, this paper addresses for the first time the problem of evaluating the effectiveness and limitations of supervised classifiers in the automatic identification of PROP taster categories (assigned to the subjects by standard non-objective scaling methods). By means of fully-automatic signal processing, event detection, waveform delineation and feature extraction, the proposed approach extracts objectively measurable morphological and dynamical features of the gustatory system activation in response to a precise taste stimulus (PROP), removing any experimenter-related variability in such processes. The gustatory system activation is measured with the same procedure described in [28], i.e. by performing a differential biopotential measurement of the depolarization of papillary cells using a custom-designed tongue electrode. The machine learning method proposed in this study could distinguish between STs and NTs with high accuracy; however, MTs were more difficult to identify. This observation could contribute to the debate in establishing the actual existence of the MTs category.

## Materials and methods

The study, approved by the Ethics Committee of the University Hospital Company (AOU Cagliari), was performed following the principles outlined in the Helsinki Declaration of 1975, as revised in 2000. All of the volunteers provided their informed consent to the protocol.

The study involved 39 healthy voluntary human subjects (16 males, 23 females, equally divided into the three PROP taster status classes), recruited within the population of post-graduate students and researchers at the Human Physiology Lab of the University of Cagliari (Italy). Exclusion criteria were the presence of known olfactory and gustatory dysfunctions, any form of disease that can compromise perceptual skills, dietary restrictions, drug use, unstable body weight, or a genotype characterized by a rare haplotype for *TAS2R38*. For women, tests were scheduled around the sixth day of the menstrual cycle to avoid taste sensitivity changes due to surging estrogen levels [29]. The preparation protocol simply entailed refraining from eating, drinking, and using oral care products or chewing gum for a minimum of 8 hours prior to testing.

The enrolled subjects underwent several physiological tests. To measure fungiform papilla density, a manual count of the papillae in the stimulation area was performed after dyeing the subjects’ dried tongues with E133 blue food dye to ease identification [11,17,30]. DNA from saliva samples were analyzed and subjects were genotyped to identify the diplotype of *TAS2R38*, categorizing them as PAV/PAV, AVI/AVI, or PAV/AVI. Genotyping was performed according to [28] using Taqman® SNP Genotyping Assays (C_8876467_10 for *rs713598*, C_9506827_10 for *rs1726866*, and C_9506826_10 for *rs10246939*) and an ABI Prism 7000 Sequence Detection System (Applied Biosystems, CA, USA) according to manufacturer specifications.

Subjects were classified as NT, MT, or ST using two different psychophysical approaches previously evaluated for validity and reliability: the three-solution test [31,32] and, after one hour, the impregnated paper screening test [11,33]. In both tests, taste intensity ratings for PROP or sodium chloride (NaCl) were provided based on the labeled magnitude scale (LMS) [31]. This scale gives subjects the freedom to rate the taste intensity for each stimulus relatively to the “strongest imaginable” oral stimulus they have ever perceived in their life. In the three-solution test, the taste intensity ratings for three supra-threshold PROP (0.032, 0.32, and 3.2 mmol/L) (Sigma-Aldrich, Milan, Italy) and NaCl (0.01, 0.1, 1.0 mol/L) (Sigma-Aldrich, Milan, Italy) solutions in water were collected, whereas the impregnated paper screening test is based on the taste intensity ratings of two paper disks, one impregnated with PROP solution (50 mmol/L) and the other with NaCl (1.0 mol/L). Subjects who gave lower intensity ratings to PROP solutions than to NaCl ones, or evaluated the PROP disk lower than 13 mm on the LMS scale, were classified as PROP NT; those who gave higher ratings to PROP solutions than to NaCl ones, or rated the PROP disk higher than 67 mm on the LMS scale were classified as ST. Finally, those who gave similar ratings to the two chemicals, or rated the PROP disk with intermediate values, were classified as MT.

Subjects who obtained different classifications by the two methods were excluded from the study. Since ST could overestimate the oral stimuli, as compared to the other taster groups [1], in order to confirm their status, they were also trained using the general labeled magnitude scale (gLMS) [34], which expands the upper limit of the scale to include sensations of any kind. The PROP taste intensity ratings were normalized based on the heaviness ratings of six opaque, sand-filled jars with masses in the range of 235–955 g [35].

### Measurement instrumental setting

The depolarization measurement after the application of PROP stimulus to the tongue was performed according to the following instrumental settings.

It was necessary to design and produce a custom electrode since no similar device was available. The electrode consisted of a 30 mm × 80 mm polyimide substrate, with a thickness of 13 μm, over which a thin film of pure silver (100 nm thick) was deposited by evaporation under high vacuum to create the active area of the electrode. The shape of this area was a ring with an outer radius of 15 mm and an inner radius of 6 mm. A 2 mm-wide silver strip, also produced by evaporation, was placed in contact with this area to transport the signal to the farthest edge of the substrate. There, an insulated copper wire, in contact with the silver strip by means of a thermosetting glue, provided a stable connection to the external measuring system. The area outside the active site of the electrode was insulated by the deposition of Parylene C, a biocompatible polymer. In order to apply the stimulus to the tongue via a paper disk impregnated with PROP solution, a circular hole was punched in the polyimide substrate to provide access to the tongue.

**Fig 1:**
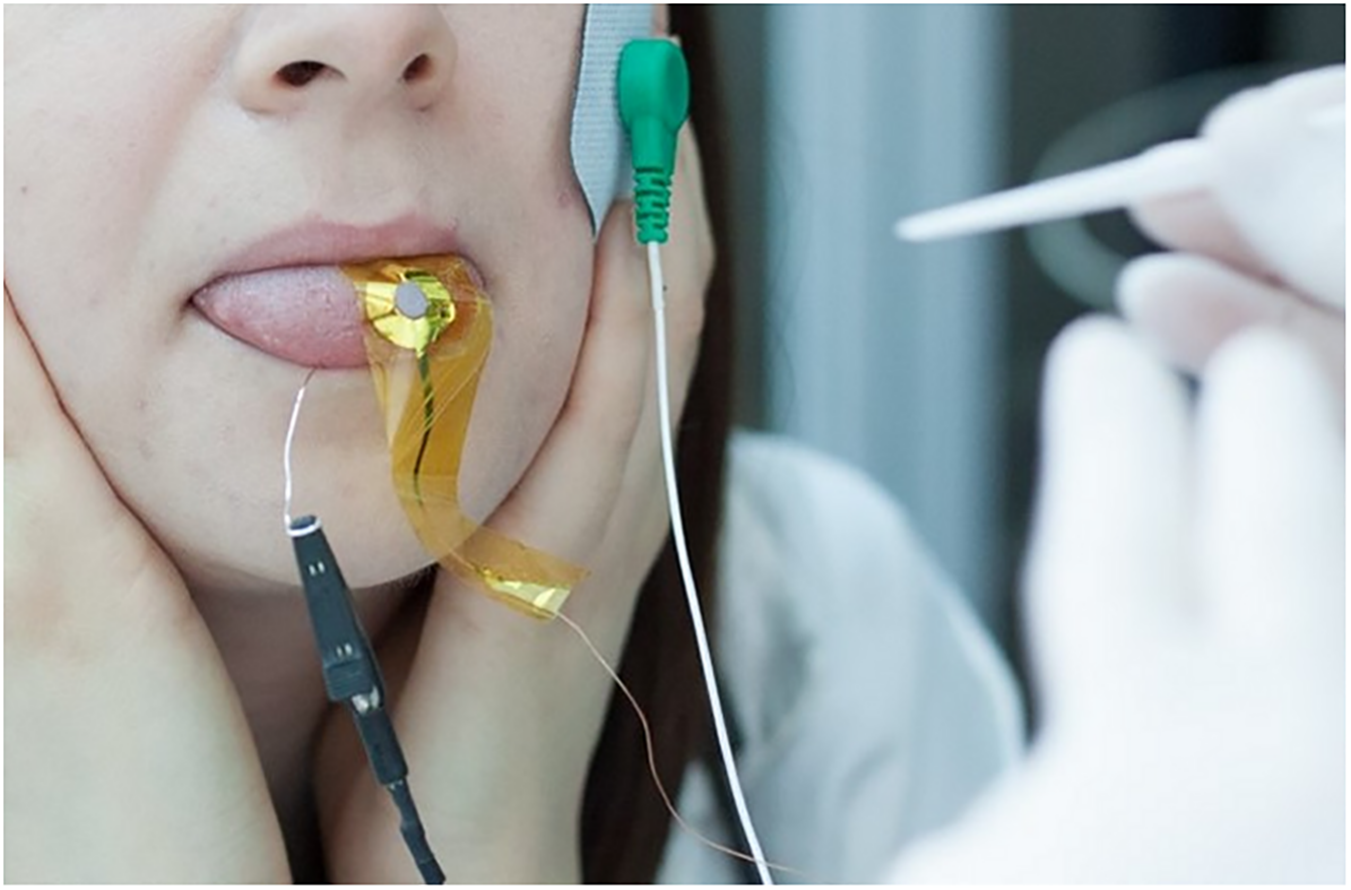
Photograph of a subject during the electrophysiological measurement. Visible in the image is the silver electrode on top of the tongue, the terminal of the silver wire rolled into a ball under the tongue, and the adhesive electrode on the cheek.

This electrode was in contact with the dorsal surface of the tongue, whereas a second electrode, consisting simply of a silver wire (0.5 mm thick) rolled into a ball, was placed on the ventral side of the tongue as a second terminal for the differential measurement. The signal ground of the measurement system was connected to the subject by means of a disposable solid hydrogel electrode (CDES003545, by SpesMedica, Italy) placed on the left cheek in order to be in an electrically neutral position with respect to the recording area. Fig 1 shows the placement of the electrodes during a test on a voluntary subject.

The recording device was a 32-channel Porti7 portable physiological measurement system (TMSI, The Netherlands), which is an isolated certified Class IIa medical device with CF-type applied parts. The electrodes were connected to the signal terminals of the AUX channels, featuring a dynamic range (±3 V) that is broader than the standard bipolar ExG channels (150 mV) in order to prevent saturation of the analog amplifier. The positive and negative inputs of the AUX channel were connected to the electrode on the dorsal and ventral sides of the tongue, respectively. The analog signals were sampled at *f*_*s*_ = 2048 Hz and digitized at 22 bits (1.43 μV resolution). At this sampling frequency, the actual bandwidth was limited by a digital decimation filter with a cut-off of approximately 550 Hz (0.27 × *f*_*s*_). The Porti7 features a medical-grade power supply and an optical fiber connection to the recording PC for improved safety and main power supply noise reduction. The recording was annotated using Polybench software (TMSI, The Netherlands).

### Electrophysiological measurement protocol

The subjects who underwent the PROP taster status classification tests using psychophysical approaches returned to the lab on a different day to undergo the same preparation protocol for the biopotential recording. The recording was performed with the subjects sitting on a chair while supporting their heads with their hands, and with tongues slightly extended out of their mouths and lips clamped shut to improve electrode stability and reduce artefacts related to the tongue musculature (Fig 1).

The depolarization of taste cells was induced by the application of a PROP stimulus that consisted of a drop of PROP solution (30 μl, 3.2 mmol/L) applied through the hole in the electrode onto the dorsal surface of the tongue by means of a filter paper disc. The recording was initiated at least 30 s prior to the stimulus application, which was indicated in Polybench, and continued for 75 s after. The stimulus was removed after 15 s, after which the electrodes were also removed. The subjects were asked to rinse their mouths with fresh water and then indicate on the LMS scale (the same used in psychophysical tests) the perceived bitterness level (62 ± 12 in STs; 29 ± 19 in MTs; 7 ± 7 in NTs). The electrophysiological signals were then converted into Matlab format for signal processing, feature extraction, and classification.

### Signal pre-processing

The recorded signals showed different wave shapes, depending on the response of the taste cells to the PROP stimuli. Due to the smooth trend of the depolarization, all signals were filtered with a low-pass equiripple finite impulse response (FIR) filter, with a cut-off frequency of 6 Hz (86^th^ order). This reduced main power supply interference and other high-frequency noise components, without affecting the main characteristics of the signal. However, due to the low-pass characteristic of the filter, the high sampling frequency of the signal was only useful for time resolution and to enable further analyses in the future.

**Fig 2:**
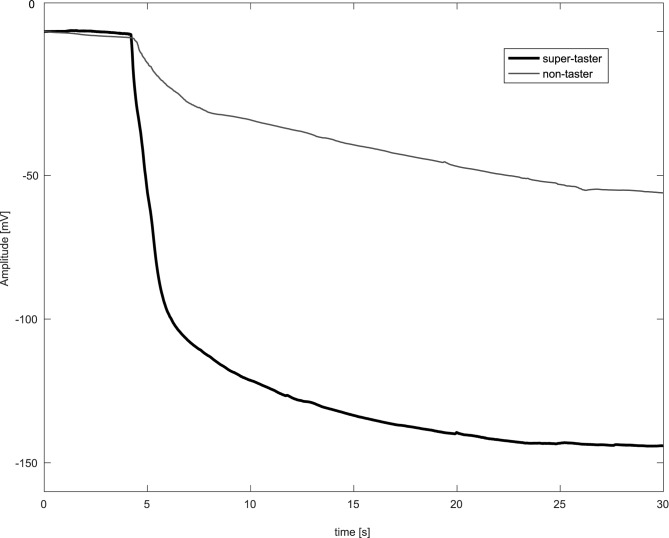
Two biopotential recordings from the tongues of a supertaster (top) and non-taster (bottom). The signals have been low-pass filtered and offset corrected to begin from zero in order to improve the clarity of the presentation.

Fig 2 presents two typical signals recorded from a ST and an NT, after application of the aforementioned low-pass filtering and an additional offset correction to improve clarity. The signals were cut in order to show the trend of the baseline 5 s before the application of the stimulus. As is seen in Fig 2, the ST exhibits a rapid change in surface biopotential, which becomes increasingly negative, until a steady level is reached. The depolarization is large in amplitude and relatively fast, which can be associated with the PAV/PAV genotype in the *TAS2R38* gene that was detected in this subject. On the contrary, the NT exhibits a much slower depolarization, with an amplitude variation of only −40 mV. Unfortunately, the presence of MTs and the variability of the wave shape hamper the correct classification of all subjects using only these two signal characteristics.

After filtering, the algorithm defined the time frame of interest, which included 5 s prior to the stimulus application and 15 s after it. To this aim, the annotations saved in Polybench during the recording were used to approximately determine the timing of these events. At this point, the sampling frequency was reduced in order to minimize processing latency and to emphasize the amplitude distance between adjacent samples. These aspects were useful for the event detection algorithm downstream. Due to the low-pass filtering of the original signal, downsampling at 128 Hz was easily obtained by retaining 1 in 16 samples, leading to the digital signal *x*[*n*].

#### Event detection

The first derivative $\dot{x}[n]$ of *x*[*n*] was used to roughly identify the end of the baseline and the beginning of the depolarization phase. The first derivative was approximated by a five-point central-difference form, which was implemented as a digital filter characterized by the following non-causal transfer function in the *z*-domain:

$$H(z)=\frac{f_{s}}{8}{[z}^{-2}+z^{-1}+z^{1}+z^{2}]$$ (1)

The algorithm then computed the moving standard deviation over the first 6 s of $\dot{x}[n]$, returning a 6-s long signal $s_{\dot{x}}[n]$, representing the local two-point standard deviation values. The knee point, representing the depolarization start time, was identified as the instant of time in which

1. $s_{\dot{x}}[n]>2.1\;mV/s$
  or
2. $s_{\dot{x}}[n]\geq 0.8\;\max\left\{s_{\dot{x}}[n]\right\}$.

The second condition was applied only when the first was not met by any point of $s_{\dot{x}}[n]$. The thresholds were empirically chosen based on the available dataset.

#### Signal approximation by curve fitting

In order to extract point features, such as the amplitudes or slopes at precise times, it is preferable to have a smooth averaged signal instead of the original one. By means of curve fitting, the depolarization curve could be simplified to obtain an analytical description useful for feature extraction.

At first, signal detrending, i.e., the subtraction of the linear trend of the signal, was adopted to reduce the gradual decrease of the biopotential over time, which was summed to the real signal [36]. However, some signals exhibited both a substantially decreasing trend in the baseline and a constant trend after the depolarization phase. In this case, detrending would result in a growth of the post-depolarization tract that was in contrast with the actual receptor physiological characteristics. For this purpose, we used linear regression to identify the signal trend before application of the stimulus (whose occurrence in time was previously detected) and after the stimulus was removed. Then,

1. if the slope of the extracted trend within the initial time interval was non-positive and the slope in the post-depolarization phase was lower than 0.2 mV/s, only an offset correction was performed such that 0 mV is achieved at the knee point;
2. otherwise, the signal trend before the stimulus application was subtracted from the entire signal.

At this point, two different regression models were used to accurately fit the detrended signal *d*[*n*]: a sum of exponential functions (2) and a rational function (3).

$$f_{e}(t)=ae^{bt}+ce^{dt}$$ (2)

$$f_{r}(t)=\frac{p_{1}t^{2}+p_{2}t+p_{3}}{t+q_{1}}$$ (3)

Such functions were able to provide a good fit for signals with different characteristics. The Matlab Curve Fitting Toolbox was used, with the tract of signal *d*[*n*] between the knee point and 15 s after (to take into account the entire depolarization) and the corresponding time array as inputs.

**Fig 3:**
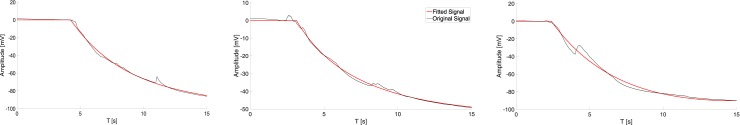
The proposed approximation by curve fitting of the detrended depolarization signal removes the artefacts in noisy signals. These artefacts are typically caused by tongue movement (when the subject has to swallow) or electrode movement. The small peak in the central plot, close to the knee point, is an artefact caused by the application of the stimulus through the impregnated paper disk.

Since the next steps were less computationally intensive and required better time resolution, at this point, the signal was resampled back to 2048 Hz by digital up-sampling and low-pass filtering, and the fiducial point related to the beginning of the depolarization was converted into the new time scale. Now, in order to approximate the completely detrended signal, the fitting functions were applied in the new time scale, and the considered values were those beginning from the first negative sample of the signal. In this way, the baseline could also be replaced by a 0-mV straight line from the beginning of the signal. On this signal, the knee point *n*_*k*_ was simply obtained by examining the crossing point between the approximated baseline and the two regression models, with a time resolution < 0.5 ms. The root mean square error between the approximated signals $\tilde{d}[n]$ and *d*[*n*], from *n*_*k*_ onward, was computed for the two regression models, indicating which model was best. Fig 3 clearly indicates how the proposed approximation of the depolarization signal using either (2) or (3) was able to correct the artefacts due to both stimulus application and tongue movements, resulting in a smoother signal for feature extraction.

Since the time frame of 15 s from the stimulus application is rather long, possible repolarization, artefacts, or noise may result in suboptimal fitting. For this reason, the depolarization end *n*_*end*_ of the signal $\tilde{d}[n]$ was estimated using the best regression model in order to perform a finer fitting between *n*_*k*_ and *n*_*end*_. For this, $\tilde{d}[n]$ was differentiated with (1) to obtain $\dot{\tilde{d}}[n]$, and then *n*_*end*_ could be:

1. the time instant in which $\dot{\tilde{d}}[n]\geq 0.02\;\max\left\{\left|\dot{\tilde{d}}[n]\right|\right\}$
  or
2. 15 s after the stimulus application.

The second condition was applied only to signals with a very slow depolarization (typically from NTs), when the first condition failed to identify any point.

#### Feature extraction

Several features were extracted in order to evaluate their ability to capture the fingerprints of the different PROP taster statuses.

Feature extraction algorithms were based on two signals:

1. the approximated signal $\tilde{d}[n]$ or its analytical form $\tilde{d}(t)$
  and
2. the signal obtained from the multiplication of the approximated signal by its first derivative, $\tilde{d}[n]∙\dot{\tilde{d}}[n]$ or its analytical form $\tilde{d}(t)\cdot \tilde{d}\prime (t)$, henceforth referred to as the feature signal.

The analytical first derivative of the approximated signal can be obtained in a closed form for (2) and (3), respectively, as

$${f\prime }_{e}(t)=abe^{bt}+cde^{dt}$$ (4)

and

$${f\prime }_{r}(t)=\frac{p_{1}t^{2}+2p_{1}q_{1}t+{{p_{2}q}_{1}-p}_{3}}{{\left(t+q_{1}\right)}^{2}}$$ (5)

Remarkably, small local changes in the signal have major consequences on its derivative, so the approximated signal provides more stable features than the detrended one.

Table 1 presents the extracted features and their descriptions. Some features represent local characteristics of either the approximated signal (2, 5) or the feature signal (6), whereas others are based on an integration procedure to gather the overall aspects of the signal rather than exact details, either of the approximated signal (3) or the feature signal (1, 4, 7).

**Table 1:** Features extracted from the depolarization signals.

Feature number	Feature name	Mathematical expression	Explanation
1	*area{d'd}*	$\int_{t_{onset}}^{t_{end}}\tilde{d}\prime (t)\tilde{d}(t)dt$	Area under the curve of the feature signal, obtained either from the sum of exponentials (2) or the rational (3) regression forms. The integration interval is considered between the beginning of depolarization (*t*_*onset*_) and its end (*t*_*end*_).
2	Δ amp	$\tilde{d}[n_{onset}]-\tilde{d}[n_{end}]$	Depolarization amplitude, i.e., the difference in mV between the values of the approximated signal $\tilde{d}\;[n]$ at *n*_*onset*_ and *n*_*end*_. Since $\tilde{d}\;[n_{\mathrm{onset}}]$ = 0 (by construction) and the signal decays (Fig 2), this is equal to the absolute value of $\tilde{d}\;[n_{\mathrm{end}}]$.
3	area{d}	$\int_{t_{onset}}^{t_{end}}\tilde{d}(t)dt$	Area under the curve of the approximated signal obtained by curve fitting (either sum of exponentials (2) or rational (3) regression forms).
4	*d'd _mean_*	$area\{\tilde{d}\prime (t)\tilde{d}(t)\}/\left(t_{end}-t_{onset}\right)$	Integral mean of the feature signal, computed as the ratio between the area under the curve of the feature signal and the depolarization interval.
5	Δ amp @2s	$\tilde{d}[n_{onset}]-\tilde{d}[n_{onset}+2f_{s}]$	Depolarization amplitude 2 s after application of stimulus. In the formula, *f*_*s*_ is the sampling frequency. Again, since $\tilde{d}\;[n_{\mathrm{onset}}]$ = 0 (by construction) and the signal decays (Fig 2), this is equal to the absolute value of $\tilde{d}\;[n_{\mathrm{onset}}+2f_{s}]$, and can be evaluated in closed form from *f*_*e*_ or *f*_*r*_.
6	max{d’d}	$max\;\left(\dot{\tilde{d}}[n]\tilde{d}[n]\right)$	Maximum value of the feature signal obtained either from the sum of exponentials (2) or rational (3) regression forms. The maximum is evaluated based on the discrete time version of the analytical signal.
7	t_1/2_	$t:\;\int_{t_{onset}}^{t}\tilde{d}\prime (\tau )\tilde{d}(\tau )d\tau =\frac{area\{d^{\prime }d\}}{2}$	Time from *t*_*onset*_ needed for the area under the curve of the feature signal to reach one half of its global value. This is obtained in closed form by analytical integration.

The analytic expression of the approximated signal and its first derivative permits the closed form exact computation of several feature values (1, 3, 4, 7), whereas other values could simply be measured from the discrete time signals used for feature extraction (2, 5) or evaluated using standard signal analysis algorithms (6). Remarkably, the feature extraction process is fully automatic, meaning that any variability in measuring a parameter due to the experimenter’s knowledge and experience is avoided, leading to repeatable and objective measurements.

#### Classification

A classification problem is a *supervised* machine learning problem where the aim is to identify which discrete category (class) a new observation belongs to by looking at the feature vector describing the observation, after a proper *training*. Training is performed by using observations labelled with the associated class, i.e. observations whose category is known [37]. Once trained, a classifier acts as a predictor for the class of unknown observations. In this work:

- classes are different on the basis of the classification problem to be solved (e.g., the three PROP taster categories, the three different diplotypes, etc.);
- observations are represented by the subjects’ depolarization signals, described by a feature vector, i.e. a vector of multiple numerical features automatically extracted from the signal by the previously described processing stages;
- features are those presented in Table 1.

During training, the mathematical model of the chosen classifier undergoes a fitting procedure on the training set of data to identify the hyper-surface in the hyper-space defined by the features, or in a different space (when kernel-based approaches are adopted), able to separate a given class from the others.

**Fig 4:**
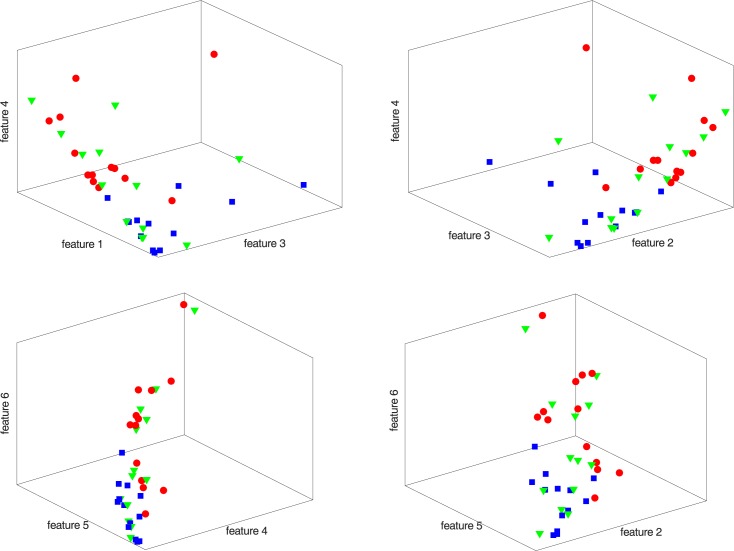
Scatter plot of the NT (blue squares), ST (red circles), and MT (green triangles) samples in the 3D space of a reduced number of features.

Feature selection is the process of finding the best feature set for a given classification problem. Features were evaluated alone and by grouping them according to the problem knowledge, until the best configuration was identified in terms of classifier accuracy. Feature combinations are presented in Table 2. Scatter plots of the features, in groups of three (Fig 4), visibly demonstrate the challenge of distinguishing between taster types due to the overlap in the feature space of the different classes.

**Table 2:** Feature combinations evaluated for the classification tests.

Combination name	Features included
Combo 1	1, 4
Combo 2	1, 2, 4
Combo 3	1, 2, 4, 5
Combo 4	1, 4, 5, 6
Best 1	1, 2, 3, 4, 5
Best 2	1, 2, 4, 5, 6
All	1, 2, 3, 4, 5, 6, 7

Three different classification problems were evaluated, two binary and one multiclass:

1. NT vs. ST
2. NT vs. Tasters (MT + ST)
3. NT vs. MT vs. ST

Furthermore, we studied another multiclass problem attempting to use the biopotential waveform to discriminate between different diplotypes relative to the *TAS2R38* gene, subdividing the dataset according to PAV/PAV, AVI/AVI, and PAV/AVI subjects.

Classification was performed by means of the Matlab Statistics and Machine Learning Toolbox (The MathWorks Inc., Natick, MA, USA). Different classifiers were evaluated in order to verify the robustness of the feature set but the best performance was obtained with k-nearest neighbors (KNN) [38], *k* = 10, either using a cosine or a cubic distance, and a support vector machine (SVM) [39] with Gaussian radial basis function (RBF) kernel, ν = 0.5, and box constraint set to 1. Since the dataset is quite small, in order to avoid overfitting, default parameters were used. The multiclass problems were solved by a one-versus-one approach [40].

The three classes, ST, MT, and NT, were represented by 13 samples each, labeled as described above. A stratified 20-times *k-fold* cross-validation scheme [41], with *k* = 10, was performed. We evaluated the mean and standard deviation of the results obtained in the different folds and graphically reported the results with box and whiskers plots. In such figures, the median is highlighted, the box demarks 50% of the samples between the first and third quartile, and the whiskers range from the minimum to the maximum value, excluding the outliers (represented with crosses). The outliers are defined as data larger than *q*_3_ + 1.5(*q*_3_ − *q*_1_) or smaller than *q*_1_ − 1.5(*q*_3_ − *q*_1_), where *q*_1_ and *q*_3_ are the 25^th^ and 75^th^ percentiles. The presence of outliers has a negative impact on the computation of the mean and standard deviation. Nevertheless, no sample was removed because of the limited dataset size.

## Results

The relationships between each feature value and the intensity of perceived PROP bitterness determined by LMS can be analyzed through a linear correlation analysis, whose results are presented in Table 3. As it can be seen, all features but three present a good level of correlation with the LMS score and, among the three, only one presents a statistically significant value of *p*. Despite this analysis surely provides some clues on the features significance, this approach is not sufficient to guarantee an adequate feature selection and the results should be used with care, since features exhibiting limited correlation with the LMS score could add information useful for the classifier to better perform the classification. In fact, the Best combos in Table 2 include either feature 3 or 6, even if they show a poor correlation with the LMS score, whilst none of them includes feature 7.

**Table 3:** Linear correlation analysis between each feature and the intensity of perceived PROP bitterness determined by LMS.

	1*	2*	3	4*	5*	6*	7
*r*	0.673	0.711	0.040	0.606	0.577	0.510	0.260
*p*	0.000007	0.000001	0.805	0.000089	0.00023	0.0014	0.123

An asterisk marks statistically significant correlations.

Classification results are divided henceforth according to the three classification problems highlighted in the previous section. Fig 4 presents the scatter plot of the different samples in the 3D space of a reduced number of features. For the first classification problem, the MT samples (green triangles) are removed, which leads to a clearly distinguishable situation. The same does not apply for the other two classification problems, in which MT samples are present (in the second one, MTs are associated with the STs). In these cases, the separability is less evident since the MT samples overlap with the NT and ST feature spaces.

### NT vs. ST

**Fig 5:**
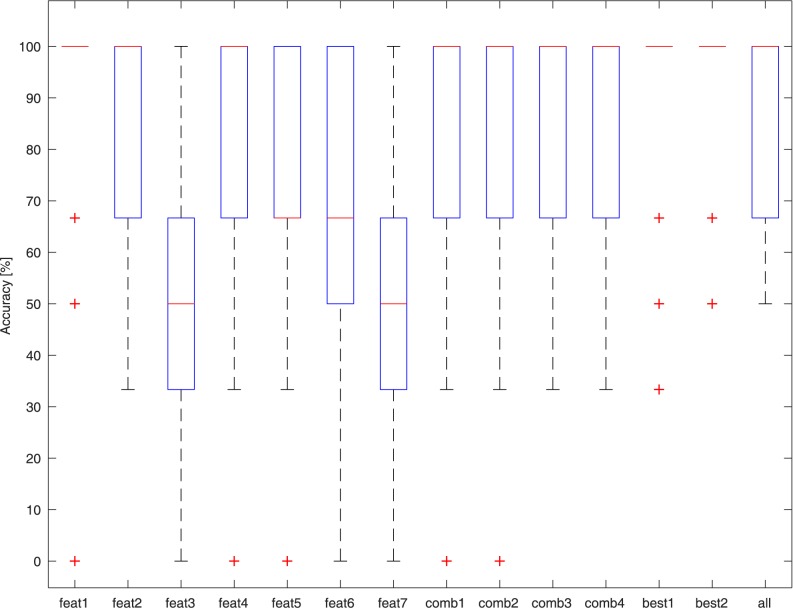
Accuracy of the Cubic KNN binary classifiers in discriminating between NT and ST samples with different feature sets.

The dataset used for this test consisted of 26 signals (13 NTs and 13 STs), and with the exception of a single ST sample that occupies the NT feature space, the other samples are easily separable. Fig 5 presents the results of the best performing classifier (Cubic KNN) on the different feature sets. Classification accuracy reached an average of 94% ± 15% with the “Best 1” feature set.

### NT vs. Tasters (MT + ST)

**Fig 6:**
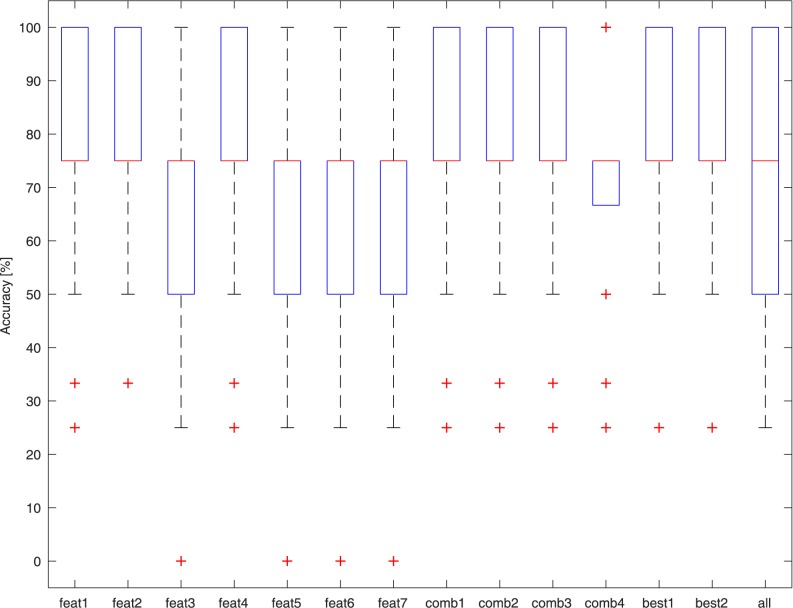
Accuracy of the Cubic KNN binary classifiers in discriminating between NT and Taster samples with different feature sets.

The dataset used for this test consisted of all 39 signals (13 NTs and 26 Tasters). Fig 6 presents the results of the best performing classifier (Cubic KNN) on the different feature sets. Classification accuracy on average reached 80% ± 18% on the “Best 1” feature set. The classification errors are attributed to the erroneous classification of some MTs as NTs.

### NT vs. MT vs. ST

**Fig 7:**
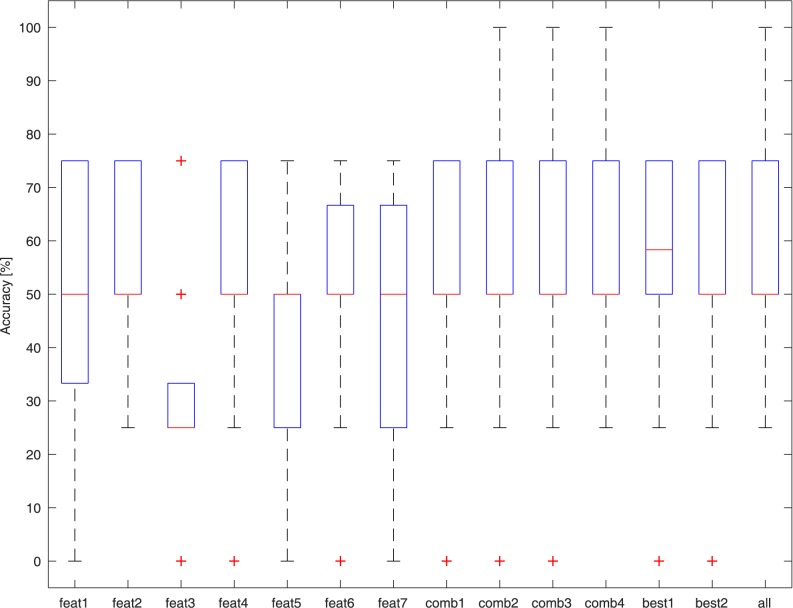
Accuracy of the Cubic KNN classifiers in discriminating between NT, MT, and ST samples with different feature sets.

Again, the dataset used for this test consisted of all 39 signals (13 NTs, 13 MTs, and 13 STs). Fig 7 presents the results of the best performing classifier (Cosine KNN) on the different feature sets. Classification accuracy on average reaches 60% ± 15% on the “Best 1” feature set. The classification errors are due to misclassification of the MT samples, which are not recognized. In fact, in this case, NTs and STs are once again correctly identified with an average accuracy of 92%.

### Comparison between KNN and SVM classifiers

**Fig 8:**
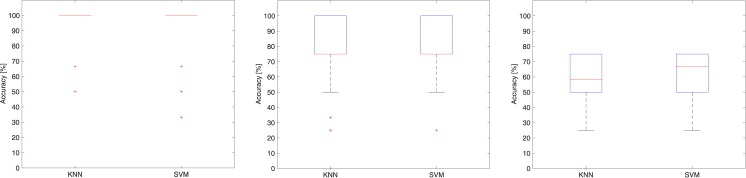
Classifiers comparisons. From left to right, comparison of the classification accuracy of Cubic KNN and SVM (left and center) and Cosine KNN and SVM (right), on the three different classification problems (NT vs. ST, NT vs. Tasters (MT + ST), and NT vs. MT vs. ST), with the associated best feature sets (Best 1 for KNN and Best 2 for SVM).

To determine whether the results were robust with respect to classifier choice, a comparison between the best performing KNN classifier on its best feature set (Best 1) and the RBF SVM classifier on its best feature set (Best 2) was performed. The results are summarized in Fig 8. Although the KNN classifier generally performed better in our tests, the SVM classifier attained similar levels of accuracy.

### Classification results for the different diplotypes

Taking into account the three classes (the two homozygotes and the one heterozygote), the same accuracy level was achieved for PROP status evaluation by the LMS scale as that achieved with the Cosine KNN on the Best 1 feature set. However, in the latter case, 70% of the heterozygote subjects were correctly classified but none of the PAV/PAV homozygote subjects were (they were erroneously assigned to the PAV/AVI class). On the contrary, the AVI/AVI variant was correctly identified.

## Discussion

The proposed method for the automatic classification of subjects as belonging to one of the three PROP taster categories reveals interesting characteristics. First, unlike the commonly used psychophysical screening methods, it is the only technique that excludes the individual subjective confounding factors of the subject under examination (the measurement on the tongue is objective) and of the experimenter (the feature extraction is fully automatic).

**Fig 9:**
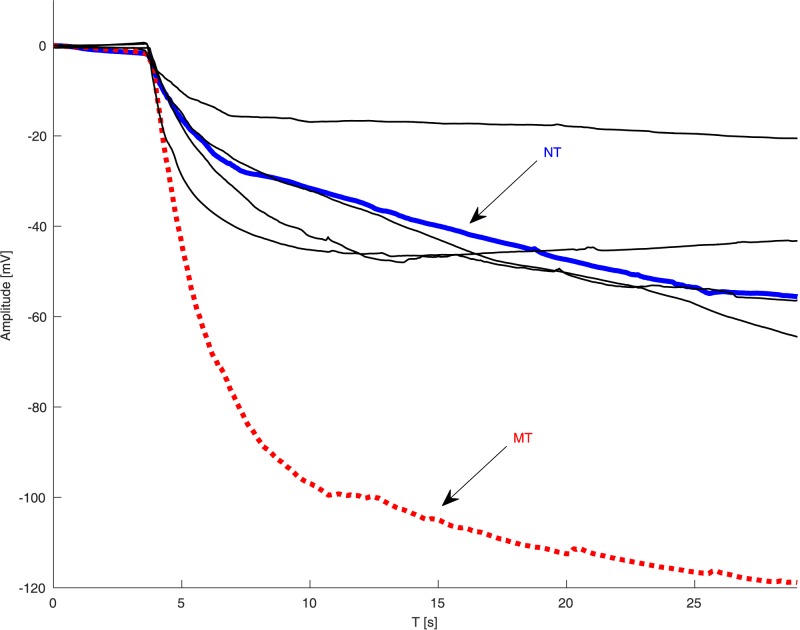
Depolarization signals of a typical MT, a typical NT, and four misclassified MTs considered as NTs.

In addition, the classification results indicate that the method is highly accurate. In fact, when only ST/NT discrimination is required, the algorithm yields up to 94% accuracy, while it reaches a reasonable 80% when MTs are included (taster/NT). In the latter case, some MTs were erroneously classified as NT by the classifier. These apparent misclassifications are actually questionable, raising the issue of labeling inaccuracies in the subjective methods, which may be intrinsic to the psychophysical approaches used in PROP taster status identification. Albeit being regularly used to classify subjects into one of the three PROP taster categories, these methods are highly subjective because subjects utilize scales to assess PROP responsiveness at higher concentrations based on their own personal experiences [23]. In this case, the bitterness ratings provided by a misclassified MT immediately after the end of the biopotential recording using the 0–100 LMS scale varied between 10 (3 subjects) and 15 (1 subject), which are closer to those given by NTs (7 ± 7), in contrast to those given by MTs (29 ± 19). From the signals presented in Fig 9, these subjects apparently exhibit a depolarization waveform closer to that of an NT than to that of an MT. On the other extreme, other MT subjects (identified according to the psychophysical test) were assigned to the ST class, which is reasonable assuming a taster/NT classification problem, which presented the highest accuracy. This is the reason for the large proportion of outliers in the boxplots presented in the Results section even for the “Best” feature sets (Fig 8). This confirms that MTs are not clearly separable from the other classes. This is in line with the analyses performed using the scatter plots in Fig 4. Remarkably, even a projection into a higher dimension feature space as performed by the SVM classifier does not help, suggesting that further studies are required to validate the actual identifiability of the MT class of subjects.

Interestingly, similar accuracies are obtained using threshold measures, reliable psychophysical procedures with a long history of use in the field that, by addressing PROP detection, can effectively separate tasters from NTs, but do not distinguish MTs from STs [1].

Likewise, *TAS2R38* genotyping can only discriminate tasters from NTs. In fact, it is known that the presence of two PAV alleles (as opposed to one) confers no additional advantage for the improved perception of bitterness from PROP stimulation [42]. This is reflected by the performance achieved by the classifier when applied to the recognition of the different diplotypes. In fact, the results confirm how the classifier was able to distinguish between two main classes of taster (PAV/PAV and PAV/AVI) and NT (AVI/AVI) subjects but could not discern any difference between the subjects presenting one PAV allele.

The robustness of the achieved results is revealed by the similar behavior of two different classifiers on the same, or similar, feature set. However, the results of this kind of classification problem are usually analyzed in terms of the ability of the classifier to correctly respect sample labels. In this case the validity of the labeling method may be questionable, as discussed above. It is then possible that the class designated by the classifiers may sometimes be more accurate than that attributed with traditional methods, thus helping to redefine the way these studies are conducted. However, even though this possibility is quite compelling, further studies are required in order to confirm this hypothesis. This is because the difficulty in distinguishing the MT class from the other classes may also be related to the low specificity of the chosen features.

Compared to the results presented in [28], this work adds a very important contribution to the research in the field by evaluating for the first time the effectiveness and limitations of supervised machine learning methods in the automatic classification of the PROP taster category of human subjects. In [28] the direct and linear correlation was shown between the amplitude and rate of depolarization signals evoked on the tongue by a PROP stimulation and: (i) the density of the fungiform papillae measured in the same area of the tongue, (ii) the intensity of the perceived bitterness, (iii) the PROP genotype and phenotype. However, the proposed analysis was not able to associate a PROP taster category to a given subject. Conversely, this paper reveals how it is possible to automatically identify the PROP taster category by simply measuring the evoked potential on the surface of the tongue in response to a PROP stimulus, with the accuracies and limitations presented above. This possibility opens new scenarios for the analysis of the PROP sensitivity compared to the psychophysical approaches traditionally used.

## Conclusion

The proposed approach utilizes a simple conformable silver electrode and an off-the-shelf biopotential measurement system approved for human use for capturing the depolarization signal fingerprints of different PROP taster statuses associated with PROP stimulation. The training of a classifier with the features extracted from the electrophysiological signals allows for distinguishing between PROP tasters and NTs very clearly. The difficulties in the identification of MTs can be considered in light of the unclear characteristics of this class, whose existence is even challenged within the scientific community. In fact, although several studies support the classification of individuals into three phenotypic groups (NT, MT and ST) [5,21,22,31,43–45], other reports suggest that PROP tasting may be a more continuous phenotype [4,14,42,46,47]. The errors of the algorithm may also reflect incorrect labeling, which was performed using the currently accepted psychophysical method, the objectivity of which is questionable. Further studies on the depolarization signal model may also improve the obtained results, achieving better performance.

The proposed approach, which is currently being extended to the investigation of other gustatory stimuli, represents the first objective and automatic method to directly measure human gustatory responses. From this perspective, it can be considered an important milestone in the study of taste function impairment and eating behavior in medicine. The method can also be applied for identifying new food products and marketing strategies in modern food sciences.
