# Correction: An automated system for the objective evaluation of human gustatory sensitivity using tongue biopotential recordings

**DOI:** 10.1371/journal.pone.0209512

**Published:** 2018-12-17

**Authors:** Danilo Pani, Ilenia Usai, Piero Cosseddu, Melania Melis, Giorgia Sollai, Roberto Crnjar, Iole Tomassini Barbarossa, Luigi Raffo, Annalisa Bonfiglio

In the Materials and methods section, there is an error in the first equation of the section titled "Event detection." Please view the complete, correct equation here:

$$H(z)=\frac{f_{s}}{8}\left[2z^{2}+z^{1}-z^{-1}-2z^{-2}\right]$$

## References

[1] Pani D, Usai I, Cosseddu P, Melis M, Sollai G, Crnjar R, et al (2017) An automated system for the objective evaluation of human gustatory sensitivity using tongue biopotential recordings. PLoS ONE 12(8): e0177246 10.1371/journal.pone.0177246 28767651 PMC5540613

